# Physiology

**Published:** 1878-07

**Authors:** 


*Colloborators:*

Dr. H. Gradle, Dr. L. W. Case, Dr. R. Park,
Dr. R. Tilley, Dr. D. R. Brower.

### Physiology.

The Gastric Juice.—(*Journal de l'Anatomie et de la Physiologie*, etc., March and April, 1878.) M. Grichet, in the above journal, has furnished its readers with a monograph on the gastric juice which cannot fail to be of great interest, both to physiologists and the medical fraternity. To appreciate the worth of the article it is necessary to know that M. G. enjoyed a privilege which happens only to few. Whilst he was serving as interne in La Pitié, a Parisian hospital, a patient obtained admission who was suffering from stricture of the œsopagus. It increased until it became absolutely impermeable. The stricture arose from taking by accident a mouthful of a strong solution of caustic potash. Its impermeability being demonstrated beyond all doubt, gastrotomy was performed by M. Verneuil, at the end of July, 1876, and in November the patient, Marcelin, was fully recovered, nourishment being of course administered by means of the gastric fistula.

M. G. has shown himself worthy of the special privilege which fell to his lot, by a series of experiments which will certainly be ranked as classical in the literature on the gastric juice. In this communication he does not confine himself entirely to his own experiments, but furnishes his readers in the first place with a succinct history of the literature on the subject, and then gives the morphology and histology of the glands of the stomach, not confining himself to the *genus homo*. It is, however, the second and third chapters that are especially interesting to the practitioner, treating respectively of: 1. The chemical constitution of the gastric juice. 2. The gastric juice mixed with the alimentary substances and the action of the gastric juice on those substances.

In reference to the chemical constitution, by collating the experiments of the most eminent experimenters, he shows the remarkable divergence of opinion as to the substance producing the acid reaction, the votes being almost equally divided in favor of lactic acid on the one hand, and hydrochloric acid on the other.

The special precaution which M. G. has taken, and which has furnished him different results, is in washing out the stomach previous to his collection of the gastric juice for analysis. It will be remembered that the œsophagus was *absolutely closed*, and that no saliva could enter. On this account, after washing out the stomach, and giving the patient something agreeable to masticate, an abundant flow of gastric juice, quite free from saliva, and otherwise quite pure, could be obtained. This is of course a *sine quâ non* of accurate analysis. It is necessary to state here that while M. G. analyzed specimens of the gastric secretion by estimation of the chlorine in different ways, he conceived the happy idea of utilizing the principle developed by Berthelot, and known as the coefficient of separation (*coefficient de partage*). The principle is this: When an aqueous solution of an acid is agitated with ether, the ether and the water divide the acid according to a constant ratio, the numerical value of which is constant for every acid.

From his experiments, carefully detailed, it results that there is present in the gastric juice absolutely no lactic acid. He summarizes the results of his first series of experiments on this subject thus:

1. Fresh and pure gastric juice contains an acid insoluble in ether, and traces of an acid which is soluble.

2. In proportion as the gastric juice becomes old (vieillit) a kind of slow fermentation develops (somewhat analagous to putrefaction) and the proportion of organic acid increases.

3. This free acid seems to be sarcolactic acid.

From a second series of experiments he concludes:

1. The acidity of the gastric juice is due to hydrochloric acid.

2. This hydrochloric acid is not in a free state. A combination similar in its various reactions can be produced by heating a solution of hydrochloric acid with an infusion of mucus from the stomach.

3. This combination is hydrochlorate of leucine.

For fear of encroaching too much, we pass by the remarks on pepsin, in order to make a few abstracts relative to the experiments made by M. G. on the relation of the gastric juice to alimentary substances. His experiments that are recorded, amount to seventy. He has paid special attention to the amount and determination of acidity of the mixed contents of the stomach; and in his experiments has always referred its acidity to hydrochloric acidity as a standard. The observations made were at intervals of from one to five hours after the introduction of the articles of food. His deductions are:

1. The acidity of the pure gastric juice gives a mean of 1.3 parts by weight of hydrochloric acid to 1,000 parts of the secretion.

2. The acidity of the gastric juice and alimentary substances taken together, gives a mean of 1.7, and seems to increase slightly toward the termination of digestion. Neither the quantity of the matter nor the liquids in the stomach seem to exercise any very perceptible influence in changing the proportion of acid.

3. Alcohol and wine increases the acidity while cane sugar diminishes it.

4. After the ingestion of acid or alkaline liquids into the stomach the general contents gradually approach the normal acidity.

To do justice to M. G.'s efforts it would be necessary to translate the whole monograph, but we must content ourselves by giving one more summation. After combating the theory that a peculiar antiseptic power is possessed by the gastric juice and showing that fermentation goes on in the stomach as well as out of it, he continues: The fermentation of the alimentary substances contained in the stomach is almost always mistaken for the secretion of the gastric juice. The exosmosis and endosmosis which is constantly taking place in the stomach, the process being facilitated by the large number of blood-vessels with which the stomach is furnished, make the acidity remain nearly always constant, varying only in a very small proportion. While the nature of the free acid, invariable for the pure gastric juice, is, on the contrary, very changeable, differing according to the nature of the food introduced. (The acid produced may be lactic, butyric, tartaric, etc.) As a certain amount of acidity seems necessary to the stomach, this development of acids seems to be a kind of economy of nature which supplements an acid secretion—the continual production of which would exhaust the system—by fermentations of different kinds. This fermentation is the more striking in proportion as the substance which produces it is more necessary to the economy, and attains its maximum of development in milk. If the gastric juice is very acid, that is, very rich in hydrochloric acid, the fermentation and putrefaction will be very low; if on the contrary the gastric is not large and not rich in hydrochloric acid, the acid fermentation and putrefaction will be very prompt.

This by no means gives a complete synopsis of M. G.'s production, but we trust sufficient has been given to afford the student points for reflection.

ANÆSTHETIC.—*Le Progrès Médical* of May 18, announces that M. Paul Bert has discovered that a mixture of 4-5 of protoxide of nitrogen and 1-5 oxygen, under an increased pressure of 1-5 of an atmosphere, will produce complete anæsthesia in a dog without any perceptible change in pulse, temperature, or respiration. The particulars of this experiment are not given, but to clothe it with authority, we need only say that Paul Bert is the successor to the renowned Claude Bernard; that he has demonstrated by experiments on himself in an air tight chamber, that the phenomena experienced by those who make high ascensions are not due, as was formerly thought, to the rarefication of the atmosphere, but to the diminution of oxygen. We cannot forbear to narrate here a little incident illustrative of M. Bert's patience as an observer: He was studying the effects of light on the sensitive plant, and during seventeen diurnal revolutions, he visited the plant night and day every two hours! The announcement of the above method of producing anæsthesia before a Parisian assembly of scientific men by a man of such patience, is not likely to be without important significance.

